# Thermal and Non-Thermal Energies for Atrial Fibrillation Ablation

**DOI:** 10.3390/jcm14062071

**Published:** 2025-03-18

**Authors:** Francesco M. Brasca, Emanuele Curti, Giovanni B. Perego

**Affiliations:** Istituto Auxologico, IRCCS Ospedale S. Luca, 20149 Milan, Italy; f.brasca@auxologico.it (F.M.B.);

**Keywords:** atrial fibrillation ablation, pulsed field ablation, cryoballoon ablation, radiofrequency ablation

## Abstract

The cornerstone of ablative therapy for atrial fibrillation (AF) is pulmonary vein isolation (PVI). Whether PVI should be added with additional lesions in persistent atrial fibrillation (PerAF) or for any post-ablative recurrent AF is a matter of debate. Whatever the ablative strategy, it must determine the choice of energy source to achieve the most durable lesion sets with the least likelihood of complications. Radiofrequency (RF) is the most studied thermal ablation technique. It can be combined with high-density electroanatomic mapping and can be used for both pulmonary and extrapulmonary atrial ablation. Cryoenergy is at least as effective as radiofrequency for PVI; it is rapid, relatively safe, and has a steep learning curve. Therefore, it has been proposed as a first-line approach for PVI-only procedures. More recently, a non-thermal technique based on the application of pulsed direct current (Pulsed Field Ablation—PFA) has been introduced. PFA causes cell death by opening cell membrane pores (electroporation) without a significant increase in tissue temperature. It is fast and does not alter the extracellular matrix as thermal techniques do, although it ends up causing long-lasting, transmural lesions. Most importantly, it is relatively selective on cardiac myocytes and therefore potentially safer than thermal techniques. Some PFA systems can be combined with electroanatomic mapping systems. However, as of now, it appears that these ablation technologies should be considered complementary rather than alternative for a number of practical and theoretical reasons.

## 1. Introduction

Catheter ablation of atrial fibrillation (AFA) has been shown to be superior to antiarrhythmic drugs (AADs) in maintaining sinus rhythm and improving symptoms and quality of life in patients with paroxysmal or persistent AF (PAF and PerAF) [1]. Better outcomes have been demonstrated not only in patients resistant to AAD treatment but also with the use of ablation as a first-line strategy [2]. Very early ablative treatment is associated with a lower likelihood of progression from PAF and PerAF to permanent AF [3]. Based on this evidence, the recent consensus statement on AF ablation by the heart rhythm societies of America, Asia, and Europe indicates AFA as the treatment of choice in symptomatic AF and AF associated with heart failure [1].

However, the generic term AF ablation encompasses a range of procedures with distinct endpoints and performed utilizing various ablative energy modalities. The purpose of this paper is to review the currently available ablative energies and how they may fit with the ablative strategies proposed to date.

## 2. Available Ablation Technologies

### 2.1. Radiofrequency

The term radiofrequency refers to an alternating electric current in the frequency range of 350 to 500 kHz. Radiofrequency current causes an increase in tissue temperature through resistive heating of the layers that are in direct contact with the tip of the ablation catheter. Passive heat conduction from this region is responsible for heating the deeper tissues. To achieve irreversible necrosis, local temperatures above 50 °C are required. If 100 °C is exceeded, clot formation and carbonization can occur on the ablation catheter, limiting energy transfer to the myocardium and the effectiveness of ablation [4]. The introduction of irrigated catheters has enabled more efficient energy delivery with larger and deeper lesions [5].

Another significant improvement in effectiveness and safety was achieved with the introduction of sensors that continuously measure contact force at the electrode–tissue interface. Force, time, power and impedance drop have been combined to develop indices to evaluate the effectiveness of individual lesions. A major issue when radiofrequency is applied to the posterior wall of the left atrium is the possibility of causing esophageal injury, which can result in an atrio-esophageal fistula, a rare but potentially fatal complication. Recently, “high-power and short-duration” (HPSD) protocols have been developed to obtain larger and shallower lesions to improve the efficiency and safety of the procedure. However, no lesion assessment algorithm has been developed for HPSD, and this limits standardization of ablation targets and reproducibility of results. To overcome this limitation, dedicated catheters with real-time tissue temperature monitoring (QDOT Micro, Biosense Webster Inc., Irvine, CA, USA; DiamondTemp, Medtronic Inc., Minneapolis, MN, USA) have been developed and tested for the delivery of short-duration high- and ultrahigh-power radiofrequency (up to 90 w for 4 s). Of note, when consecutive lesions are administered in the posterior wall in close proximity to each other, the phenomenon of “heat stacking” could lead to equally penetrating lesions [6]. On the other hand, HPSD might be associated with a higher rate of asymptomatic cerebral microembolism [7].

A multi-electrode RF balloon catheter (HELIOSTAR, Biosense Webster Inc., Irvine, CA, USA) has also been developed for single-shot PV deconnection, equipped with a multiple electrode array and compatible with the CARTO mapping system. Evidence on efficacy and safety is only preliminary and observational [8].

Finally, a catheter (Sphere-9, Affera Inc., Newton, MA, USA) for focal radiofrequency ablation with a “large-footprint” lattice tip, which causes larger lesions, has been developed, convenient for making circumferential point-to-point lesions around the antra of PVs. The ablation catheter is coupled to a dedicated mapping system and can switch to PFA [9].

Radiofrequency has specific complications, the incidence of which has been reduced as technologies and procedures have evolved. When PVI is achieved by antral lesion lines, pulmonary vein stenosis will occur in less than 1% of cases [10]; cardiac tamponade can occur either as a result of catheter manipulation or radiofrequency perforation in 0.3–0.8% of patients. Esophageal injury is a rare (0.07%) but potentially fatal event. The introduction of HPSD should reduce its likelihood by decreasing the penetration of energy into tissues contiguous to the atrial wall [11].

### 2.2. Cryoablation

Cryoenergy was first applied for PVI in 2002, but it was not until 2007 that the first cryogenic balloons were used on humans. When the temperature of myocardial tissue is reduced, an initial phase of reversible cellular dysfunction occurs. Later (with a threshold of about −25 °C), the injury becomes irreversible: ice crystals form in the cytoplasm, with a dysfunction of organelles and membranes, and this effect is further exacerbated during the rewarming phase, when crystals expand before fully melting. Subsequently, hemorrhage and inflammation occur, and finally, the healing process leads to the formation of fibrotic tissue. Compared with radiofrequency, cryoablation causes less endothelial damage, and the final scars are denser, with more defined borders and preserved tissue matrix. Therefore, cryoablation is expected to be less proarrhythmic and thrombogenic [12,13].

**Figure 1:**
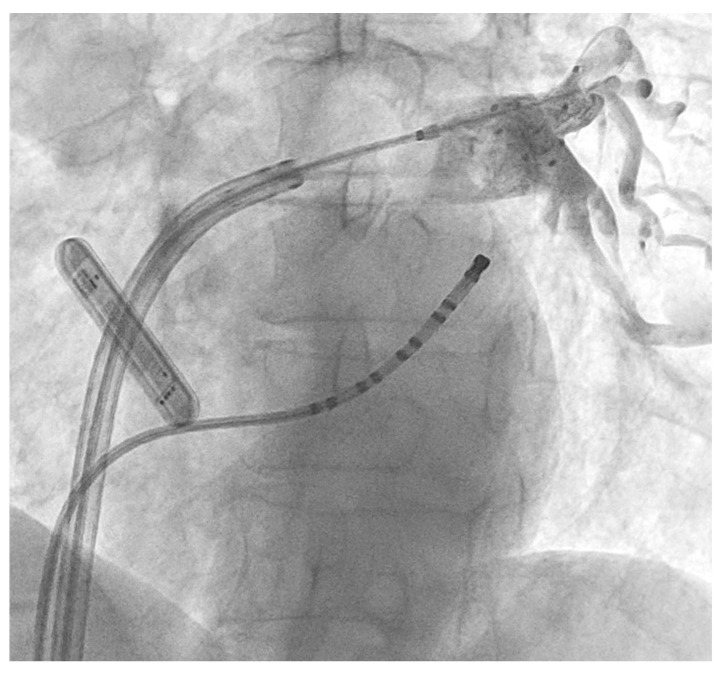
Arctic Front^TM^ cryoballoon ablation catheter. Contrast injection in left inferior pulmonary vein.

Two manufacturers have developed commercially available devices. The Arctic Front^TM^ cryogenic balloon (Medtronic, Minneapolis, MN, USA) consists of an outer balloon and an inner balloon, with coolant (NO) circulating in the inner balloon and thermocouples measuring the temperature inside the balloon (Figure 1).

A second-generation balloon had a significantly larger freezing zone to improve efficacy.

The POLARx (Boston Scientific, Natick, MA, USA) cryoballoon catheter, which received approval in Europe in 2023, is quite similar to the first, but has conformable dimensions intended to better fit different-sized veins.

Both systems are designed to deliver cryoenergy exclusively to the antral zone of pulmonary veins, although off-label use at other sites (cavo-tricuspid isthmus and left atrial roof) has been reported. When used for approved indications, cryoenergy has been shown to be relatively safe. The most frequent complication is transient phrenic paralysis (PNP), with an incidence of about 3% (second-generation balloons). Care should be taken to avoid right phrenic injury by constantly monitoring nerve conduction: with these precautions, phrenic paralysis is temporary in most cases [14]. Isolated cases of esophageal damage and PV stenosis have been reported if energy is delivered too deep into the veins [1].

### 2.3. Pulsed Field Ablation

The earliest experiences with the use of electric fields for cardiac ablation date back to the 1980s, when Scheinman et al. used DC shock delivered by a defibrillator to achieve atrioventricular node blockade [15]. The procedure, although effective, was associated with rapid tissue heating and vaporization and barotrauma. Current PFA involves delivering a series of repeated, short-duration discharges that have minimal thermal effect but result in the accumulation of charges on the two sides of the cell membrane, which acts like a capacitor. As a result, the phospholipid molecules that constitute the cell membrane re-orient themselves to form physical channels (nanopores), which make the membrane permeable to water. If the applied voltage is of sufficient magnitude and duration, alterations in membrane integrity and function occur, impairing the maintenance of cellular homeostasis due to the loss of electrical and concentration gradients, and “electroporation” becomes irreversible, causing immediate cell death. Furthermore, over 2–7 weeks, an additional proportion of myocardial cells are lost through apoptosis, resulting from an altered cellular pH, the generation of free radicals, and the release of mitochondrial cytosome C [16]. In the following 6 to 8 weeks, the maturation of the lesion is completed with the establishment of a fibrous scar, with no alteration of the vascular and tissue structure. PFA differs from thermal methods in some relevant aspects:

- The extent and characteristics of the lesion obtained depend on factors such as voltage, pulse amplitude, waveform, polarity, and physical shape of the catheter, which cannot be subject to adjustment by the end user and are strictly dependent on the system used. Efficacy and lesion geometry are therefore specific, and results obtained with individual systems are not generalizable.
- PFA results in an increase in tissue temperature due to the Joule effect (resistive heating), although it does not contribute to lesion formation [17]. Verma et al. report muscle temperature changes of less than 2.8 °C at a depth of 3 mm [18]. Although small, this temperature increase at the catheter tip can be used to monitor energy transfer to the tissue during energy delivery for optimal lesion formation. Indeed, the use of an esophageal probe [19] or techniques such as endoscopy, endoscopic ultrasound, and electrogastrography before and after PVI have confirmed insignificant rises in temperature and no tissue damage [20].
- Thermal methods act nonspecifically on tissues. In contrast, sensitivity to PFA is extremely different for different tissues. Heart muscle cells are particularly vulnerable to PFA, three times more so than the esophageal wall and four times more so than the phrenic nerve [21]. In addition, even thin layers of adipose tissue, such as those separating the posterior wall of the atrium from the esophagus, act as insulators against PFA. For these reasons, the introduction of PFA has been accompanied by positive expectations regarding safety [22]. Indeed, in clinical and preclinical trials, the use of PFA has not been associated with long-term esophageal injury [23,24].

On the other hand, electroporation has specific side effects:

- It can result in skeletal muscle contraction (more frequent for unipolar systems), which requires adequate sedoanalgesia (although in most studies, paralytics are not necessary) [25,26].
- Vasospasm can occur when pulsed fields are applied near coronary vessels, although there are no published data demonstrating long-term effects on coronary arteries. To overcome this problem, intracoronary nitrate administration has been used during ablation [27,28].
- PFA is associated with the formation of microbubbles, likely due to the release of nitrogen in the gaseous state. The small size of the bubbles (<40µ) and their composition would allow for their rapid reabsorption and explain the low incidence of silent cardioembolic events on MR [29].
- PFA can induce cough, even in patients under general anesthesia, by direct stimulation of pulmonary J receptors.

## 3. Technology Application to Ablation Strategies

In PAF, the initial ablative treatment of choice is pulmonary vein isolation (PVI). For all other cases (PAF redo procedures, PerAF, and long-standing PerAF), there is no shared consensus on ablative strategies, which can be summarized as follows [1]:

- PVI;
- Atrial lines (CT isthmus, mitral isthmus, anterior mitral line, left atrial roof, posterior wall isolation);
- Debulking of the posterior wall;
- Substrate ablation, targeting low voltages, areas of slow conduction, fragmented potentials.

Hereunder, we will analyze the merits and shortcomings of individual technologies in these specific contexts.

### 3.1. Pulmonary Vein Isolation

Early PVI trials were carried out with first-generation radiofrequency, soon replaced by irrigated catheters. The introduction of contact force monitoring and indices for the real-time assessment of lesion quality significantly improved the efficacy and duration of point-by-point isolation of pulmonary veins with radiofrequency and reduced the recurrence rate in PAF.

According to observational studies, HPSD radiofrequency ablation substantially improved procedural efficiency, with an even lower risk of arrhythmic recurrence at 12 months [30]. Small RCTs have demonstrated a lower incidence of arrhythmias at 12 months as compared to low-power RF [31,32].

The STOP AF study first assessed the safety and efficacy of CB PVI in PAF, raising major concerns about the high rate of temporary phrenic nerve palsy (11.2%). Subsequently, several studies in which larger diameter balloons were systematically used were conducted, showing a much lower rate of this complication. In addition, several authors have evaluated the duration of injury by repeated mapping, and comparisons have been made between CB and RF.

A meta-analysis by Seban et al. [30] was able to show only a nonsignificant trend in favor of CB, in terms of lesions durability, with a very wide dispersion of RF performance, confirming a higher operator dependence of this method.

Balloon cryoablation and point-by-point radiofrequency PVI were directly compared in the FIRE AND ICE [33] study, which was able to demonstrate the noninferiority of cryoablation with regard to the recurrence of atrial arrhythmias, need for treatment with antiarrhythmic drugs, and repeat ablation at one year. In fact, the recurrence of atrial arrhythmias was only non-significantly lower for cryoballoon but reached statistical significance when events occurring in the first three months were included, although this secondary analysis has been widely criticized on methodological grounds [29]. The procedural time was shorter for cryoablation, while the fluoroscopic time was shorter for the RF group. Temporary and permanent phrenic nerve palsy was observed in 2.7% and 0.3% of cryoablation cases, respectively. More recently, the CIRCADOSE study compared last-generation CB technology with RF contact force catheters. No difference was found in the recurrence of atrial arrhythmias at 1 year, but both thermal energies achieved a 99% reduction in AF burden [34].

Direct comparisons between HPSD and cryogenic balloon ablation have not yet been published.

Several systems for PFA have been used for PVI. Table 1 summarizes the most relevant technical features and published evidence. 

It has been shown that the reconnection rate of PVs at the time of remapping is lower for PFA than for thermal energies [40]. However, no single RCT study has been able to demonstrate a statistically significant reduction in clinical recurrence at 1 year after PFA compared with cryoballoon or radiofrequency ablation (Table 2). Further and possibly larger studies are needed to ascertain whether PFA meets expectations for improved clinical outcomes after isolated PVI procedures in PAF.

### 3.2. Linear Lesions

Based on the favorable experiences obtained with surgical ablation (the Cox maze procedure), several attempts have been made over the years to reproduce the same results by the endocardial route, with mono- or biatrial linear lesions. Lines were most commonly placed at the roof of the left atrium, at the mitral isthmus (between the valve ring and the left inferior pulmonary vein), and at the cavo-tricuspid isthmus. In addition to these, intercaval, anterior mitral, and tailored lines were proposed to be anchored to pre-existing scar areas. All this was accomplished with radiofrequency under the guidance of mapping systems. However, no randomized trial has ever demonstrated an incremental benefit of linear ablation in PAF or PerAF. On the other hand, the proarrhythmic potential of incomplete linear lesions, which are often the result of these ablative strategies, has been proven [47,48].

However, linear lesions have a primary role in the ablation of atrial macro-re-entrant arrhythmias that are associated with AF or may indeed be a consequence of the initial ablative treatment [49,50].

It was hypothesized that the inability to reproduce surgical results endocardially was due to the difficulty of RF in obtaining effective transmural and persistent lesions over time. It was therefore hypothesized that PFA may reopen the possibility of using linear ablation for complex substrates or in PerAF ablation. The only PFA catheter currently capable of performing point-to-point linear lesions is the Sphere 9 (Affera Inc., Newton, MA, USA).

With this system, linear PFA lesions were shown to be very safe (0.6% minor complications and no significant increase in esophageal temperature) in a mixed population of patients with PAF and PerAF [51]. Invasive remapping demonstrated the durability of PVI in 97% of PVs and of all linear lesions in 91% of cases [9]. The SPHERE Per-AF study demonstrated the non-inferiority of large-footprint AF ablation to radiofrequency when linear lesions were added for ablation of PerAF. In fact, freedom from recurrence in a single procedure was as high as 73%, although the difference from radiofrequency did not reach statistical significance [52].

### 3.3. Debulking or Isolation of the Posterior Wall

The left atrial posterior wall has the same embryological origin as PVs and, like the latter, is densely innervated by parasympathetic plexi [53,54]. This raised the hypothesis that it may play an arrhythmogenic role in AF and that its electrical deactivation (Posterior Wall Isolation—PWI) may contribute to rhythm control.

Several sets of linear lesions and extended ablation (debulking) of the posterior wall have been proposed for this purpose, but the results of clinical studies are conflicting [1]. In most of them, PWI was performed with radiofrequency, with limitations posed by the need to avoid heating of the esophagus. This element, in particular, could explain the inconsistency of the results and justify the use of other energy sources to achieve deeper and truly transmural lesions.

Therefore, PWI was performed with the cryoballoon, although completion with RF was necessary in up to 45% of cases [55]. 950. Extensive posterior wall debulking was also achieved with the pentaspline PFA catheter. After 3 months, PWI persisted in 100% of patients [56]. Further evidence is needed to assess whether the greater persistence of PWI achieved with PFA is associated with better clinical outcomes.

### 3.4. Substrate-Guided Ablation

Atrial fibrosis is a hallmark of atrial cardiomyopathy and plays a central role in the pathogenesis of AF, contributing to its onset and progression [57].

MRI provides a safe and noninvasive method to detect atrial fibrosis in patients with AF. The burden of fibrosis detected by MRI is directly related to the risk of recurrence [58]. On the other hand, HD-EAM can identify areas of low voltage (LV), fractionated potentials, and areas of slow conduction that are considered surrogate markers of fibrosis. In fact, local atrial voltages below a threshold of 0.7–0.5 mV are associated with LGE on MRI [7,8].

Under the assumption that ablation targeting focal fibrosis can improve clinical outcomes, MRI has been proposed as a guide to ablation, but this approach has proven ineffective [59]. Low-voltage guided ablation has yielded conflicting results.

Several RCTs, all of which use radiofrequency as an energy source, have provided conflicting evidence on AFA targeted to low-voltage areas. In the VOLCANO and the STABLE-SR II studies, the addition of low-voltage ablation to PVI did not improve outcomes in PAF. According to the STABLE-SR-III study, the ablation of low-voltage areas in PAF conferred a significant incremental benefit [60].

The ERASE AF trial randomized atrial fibrillation patients to PVI alone or PVI plus substrate modification, performed only in patients with low-voltage areas on high-density electroanatomic mapping in sinus rhythm. The recurrence rate was significantly lower in substrate-guided ablation [61]. Finally, two systematic reviews and meta-analyses evaluated randomized controlled trials that compared fibrosis-guided ablation using LVA or LGE-CMR [62] or LVA alone [63] with conventional PVI. Both showed that fibrosis-guided ablation significantly reduced the recurrences of atrial arrhythmia.

Note that all studies on fibrosis-guided ablation have used radiofrequency as an energy source. Indeed, there is direct evidence that local atrial scarring may hinder the effectiveness of radiofrequency in the pulmonary vein [64]. In fact, several experimental animal models have shown that PFA is more effective than radiofrequency in achieving transmural injury, where the scar protects deeper layers of healthy ventricular myocardium, and in border regions [65,66]. Cases have been reported in which PFA was able to achieve successful ablation of complex atrial arrhythmias where radiofrequency had previously failed.

Thus, it can be speculated that PFA may be a more effective ablation technique than radiofrequency when adopting a fibrosis-guided strategy [67].

To date, no studies of substrate-guided PFA have been published.

## 4. Limitations

Although the volume of scientific evidence on PFA is rapidly growing, for now, it is not comparable with that available for RF and cryoablation.

The follow-up of PFA procedures is still short, not superior to 12 months, and shorter than what is available for other energy sources. Long-term data and real-world results should be investigated to allow for stronger recommendations about energy source use in arrhythmia ablation.

Events taking place in the first 3 mos. after AFA might be predictive of recurrence, differently from what has been observed for thermal energies. According to this observation, a shorter blanking period of 1 month has been proposed for PFA ablation follow-up, which might lead to a reinterpretation of the results of the first clinical trials [68].

## 5. Future Directions

Much of what is presented in this paper is based on the assumption that PFA should mimic the same lesion patterns developed for cryoablation and radiofrequency ablation.

However, PFA lesions are more durable and transmural and can be applied with differently shaped instruments. Therefore, it cannot be excluded that different ablation strategies will be developed specifically for this new technology or that more established strategies will lead to significantly different results with a different energy source. This could be the case for extrapulmonary lesions in persistent AF, guided by voltage or applied in a standard pattern (posterior wall, roof, mitral, anterior lines). The relative independence of PFA transmurality from fibrosis could lead to more favorable and uniform clinical outcomes than previously reported with radiofrequency.

Artificial intelligence could add further complexity and allow for an even more personalized approach to ablation for the patient but will still target fibrosis markers and need more effective ablation energies.

A stepwise approach with PVI as first-line treatment, implemented with dedicated one-shot instruments, could be a reasonable and cost-effective solution. For PVI-only procedures, any energy would be suitable, although preliminary evidence suggests that cryo might be better than RF but inferior to PFA [40]. Any further ablative steps would require versatile instruments capable of achieving linear or focal lesions. There is a potential for PFA to become the energy of choice for this second stage with this approach.

## 6. Conclusions

For the time being, radiofrequency, cryoablation, and PFA all maintain specific roles in the ablative technique landscape, depending on the ablative strategy to be applied in individual cases. PFA may be relatively safer than the other techniques, but although the volume of scientific evidence on PFA is rapidly growing, for now, it is not comparable with that available for RF and cryoablation, and it is too early to assume that it is destined to establish itself as the technique of choice.

## Figures and Tables

**Table 1:** Commercially available PFA systems and supporting evidence.

Farapulse™ (by Boston Scientific, Natick, MA, USA) 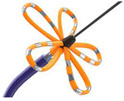 Bipolar penta spline catheter	It proved effective in terms of lesion persistence 641, with a 1-year arrhythmia-free survival of 78% in PAF. Two multicenter registries (MANIFEST-PV and EU-PORIA) gave similar results in a mixed population of PAF and PerAF [35]. The same device was evaluated in the randomized ADVENT trial, which indicated the noninferiority of PFA to both contact force RF and latest-generation cryoballoon ablation [36].
PulseSelect^TM^ (by Medtronic Inc., Minneapolis, MN, USA) 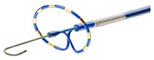 Circular over-the-wire	In the PULSED AF study, the PulseSelect^TM^ System achieved a 100% acute success rate for PVI with essentially no acute complications and a 1-year recurrence rate comparable to that of RF [37].
Varipulse VLCC™ (by Biosense Webster Inc., Irvine, CA, USA) 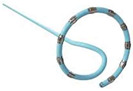 Variable loop biphasic circular catheter	VLCC^TM^ obtained 71% freedom from atrial arrhythmias at 1 year in PAF, with no procedure-related adverse events [38].
Sphere9^TM^ (by Medtronic Inc., Minneapolis, MN, USA) 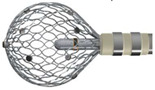 Large-footprint lattice catheter	Sphere9^TM^, delivering both radiofrequency and PFA, achieved 78% freedom from atrial arrhythmias at 1 year in a mixed population of PAF and PerAF [9].
Sphere 360^TM^ (by Medtronic Inc., Minneapolis, MN, USA) 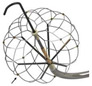 Lattice PVI-only single-shot	Currently under development. Preliminary data suggest an efficacy of PVI, with a 45-day isolation durability, of up to 99% [39].

**Table 2:** Comparison of PFA to cryoballoon and RF ablation for PAF [36,40,41,42,43,44,45,46].

Study	*n*	Endpoint	Freedom from Endpoint (at 12 mos.)	*p*	Procedure Duration (min)	*p*	Fluoroscopy Time (min)	*p*	Complications	*p*
***PFA vs. Thermal (Cryo and RF)***										
Reddy et al. [39] ADVENT	305 PFA 302 thermal	Recurrence of AT/AF or AADs or repeat TCA	73.1% 71.3%	n.s.	105.8 ± 29.4 123.1 ± 42.1	<0.05	21.1 ± 11.0 13.9 ± 12.8	<0.05	2.0% 1.3%	n.s.
Della Rocca et al. [40] (HRMC trial)	174 PFA 348 Cryo 348 RF	AF/AT Recurrence	79.3% 74.7% 72.4%	n.s.	52.1 ± 14.6 64.5 ± 21.8 84.8 ± 24.8	<0.001	14.8 ± 3.4 17.6 ± 8.1 12.9 ± 6.9	<0.001	1.1% (*) 1.1% (*) 0.9% (*)	n.s.
Maurhofer et al. [41]	40 PFA 80 Cryo 80 RF	AF/AT Recurrence	85.0% 76.8% 66.2%	n.s.	93 (79–116) 75 (60–97) 182 (134–23)	<0.001	26 (21–31) 17 (13–24) 7 (3–13)	<0.001	1 (**) 0% 0%	<0.04
***PFA vs. cryo***										
Badertscher et al. [42]	106 PFA 75 Cryo	AF/AT Recurrence	76% 70%	n.s.	55 (43–64) 58 (48–69)	0.09	11 (9.3–14) 11 (8.7–16)	n.s.	2.8% 4%	n.s.
Rattka et al. [43]	94 PFA 47 Cryo	AF/AT Recurrence	70% 61%	n.s.	162 ± 64 163 ± 62	n.s.	26 ± 9 23 ± 9	0.06	4.2% 2.1%	n.s.
Schipper et al. [44]	54 PFA 54 Cryo	AF/AT Recurrence	74% 72%	n.s.	64.5 ± 17.5 73.0 ± 24.8	0.07	15.3 ± 4.7 12.3 ± 5.3	n.s.	3.7% 11%	n.s.
Urbanek et al. [45]	200 PFA 200 Cryo	AF/AT Recurrence	74% 78%	n.s.	34.5 (29–40) 50 (45–60)	<0.001	7.1 (5.5–8.9) 6.9 (5.5–8.8)	n.s.	6 13	n.s.
***PFA vs. RF HPSD***										
Reinsch et al. [46] (PRIORI study)	201 PFA 210 RF	AF/AT Recurrence	85% 79%	n.s.	61 (44–103) 125 (105–143)	<0.001	16 (13–20) 4 (2–5)	<0.001	3% 6.2%	n.s.

Procedure and fluoroscopy times are reported as average ± standard deviation or as median and interquartile range. *p* is indicated only if <0.1. (*) only major complication. (**) cardiac tamponade.

## References

[1] Tzeis S., Gerstenfeld E., Kalman J., Saad E., Shamloo A., Andrade J., Barbhaiya C., Baykaner T., Boveda S., Calkins H. (2024). 2024 European Heart Rhythm Association/Heart Rhythm Society/Asia Pacific Heart Rhythm Society/Latin American Heart Rhythm Society expert consensus statement on catheter and surgical ablation of atrial fibrillation. Europace.

[2] Kirchhof P., Camm A.J., Goette A., Brandes A., Eckardt L., Elvan A., Fetsch T., van Gelder I.C., Haase D., Haegeli L.M. (2020). Early Rhythm-Control Therapy in Patients with Atrial Fibrillation. N. Engl. J. Med..

[3] Kuck K.H., Lebedev D.S., Mikhaylov E.N., Romanov A., Gellér L., Kalējs O., Neumann T., Davtyan K., On Y.K., Popov S. (2021). Catheter ablation or medical therapy to delay progression of atrial fibrillation: The randomized controlled atrial fibrillation progression trial (ATTEST). Europace.

[4] Nath S., Haines D.E. (1995). Biophysics and pathology of catheter energy delivery systems. Prog. Cardiovasc. Dis..

[5] Michaud G.F., Kumar S. (2016). Eliminating Coagulum Formation with Charge Delivery During Radiofrequency Ablation Negative May Be a Positive!. JACC Clin. Electrophysiol..

[6] La Fazia V.M., Pierucci N., Schiavone M., Compagnucci P., Mohanty S., Gianni C., Della Rocca D.G., Horton R., Al-Ahmad A., Di Biase L. (2024). Comparative effects of different power settings for achieving transmural isolation of the left atrial posterior wall with radiofrequency energy. Europace.

[7] Lee A.C., Voskoboinik A., Cheung C.C., Yogi S., Tseng Z.H., Moss J.D., Dewland T.A., Lee B.K., Lee R.J., Hsia H.H. (2023). A Randomized Trial of High vs Standard Power Radiofrequency Ablation for Pulmonary Vein Isolation: SHORT-AF. JACC Clin. Electrophysiol..

[8] Reddy V.Y., Schilling R., Grimaldi M., Horton R., Natale A., Riva S., Tondo C., Kuck K.H., Neuzil P., McInnis K. (2019). Pulmonary Vein Isolation with a Novel Multielectrode Radiofrequency Balloon Catheter That Allows Directionally Tailored Energy Delivery: Short-Term Outcomes from a Multicenter First-in-Human Study (RADIANCE). Circ. Arrhythm. Electrophysiol..

[9] Reddy V.Y., Peichl P., Anter E., Rackauskas G., Petru J., Funasako M., Minami K., Koruth J.S., Natale A., Jais P. (2023). A Focal Ablation Catheter Toggling Between Radiofrequency and Pulsed Field Energy to Treat Atrial Fibrillation. JACC Clin. Electrophysiol..

[10] Schoene K., Arya A., Jahnke C., Paetsch I., Nedios S., Hilbert S., Bollmann A., Hindricks G., Sommer P. (2018). Acquired Pulmonary Vein Stenosis After Radiofrequency Ablation for Atrial Fibrillation: Single-Center Experience in Catheter Interventional Treatment. JACC Cardiovasc. Interv..

[11] Nair K.K., Shurrab M., Skanes A., Danon A., Birnie D., Morillo C., Chauhan V., Mangat I., Ayala-Paredes F. (2014). The prevalence and risk factors for atrioesophageal fistula after percutaneous radiofrequency catheter ablation for atrial fibrillation: The Canadian experience. J. Interv. Card. Electrophysiol..

[12] Khairy P., Chauvet P., Lehmann J., Lambert J., Macle L., Tanguay J.F., Sirois M.G., Santoianni D., Dubuc M. (2003). Lower incidence of thrombus formation with cryoenergy versus radiofrequency catheter ablation. Circulation.

[13] Mazur P. (1984). Freezing of Living Cells: Mechanisms and Implications. Am. J. Physiol.-Cell Physiol..

[14] Guhl E.N., Siddoway D., Adelstein E., Bazaz R., Mendenhall G.S., Nemec J., Saba S., Schwartzman D., Voigt A., Wang N.C. (2016). Incidence and Predictors of Complications During Cryoballoon Pulmonary Vein Isolation for Atrial Fibrillation. J. Am. Heart Assoc..

[15] Scheinman M.M., Scheinman M.M. (1982). Catheter-induced ablation of the atrioventricular junction to control refractory supraventricular arrhythmias. JAMA J. Am. Med. Assoc..

[16] Xie F., Varghese F., Pakhomov A.G., Semenov I., Xiao S., Philpott J., Zemlin C. (2015). Ablation of myocardial tissue with nanosecond pulsed electric fields. PLoS ONE.

[17] Sugrue A., Maor E., Del-Carpio Munoz F., Killu A.M., Asirvatham S.J. (2022). Cardiac ablation with pulsed electric fields: Principles and biophysics. Europace.

[18] Verma A., Zhong P., Castellvi Q., Girouard S., Mediratta V., Neal R.E. (2023). Thermal Profiles for Focal Pulsed Electric Field Ablation. JACC Clin. Electrophysiol..

[19] Kirstein B., Heeger C.H., Vogler J., Eitel C., Feher M., Phan H.L., Mushfiq I., Traub A., Hatahet S., Samara O. (2024). Impact of pulsed field ablation on intraluminal esophageal temperature. J. Cardiovasc. Electrophysiol..

[20] Grosse Meininghaus D., Freund R., Koerber B., Kleemann T., Matthes H., Geller J.C. (2024). Pulsed-field ablation does not induce esophageal and periesophageal injury—A new esophageal safety paradigm in catheter ablation of atrial fibrillation. J. Cardiovasc. Electrophysiol..

[21] Tabaja C., Younis A., Hussein A.A., Taigen T.L., Nakagawa H., Saliba W.I., Sroubek J., Santangeli P., Wazni O.M. (2023). Catheter-Based Electroporation: A Novel Technique for Catheter Ablation of Cardiac Arrhythmias. JACC Clin. Electrophysiol..

[22] Maor E., Ivorra A., Rubinsky B. (2009). Non thermal irreversible electroporation: Novel technology for vascular smooth muscle cells ablation. PLoS ONE.

[23] Stewart M.T., Haines D.E., Miklavčič D., Kos B., Kirchhof N., Barka N., Mattison L., Martien M., Onal B., Howard B. (2021). Safety and chronic lesion characterization of pulsed field ablation in a Porcine model. J. Cardiovasc. Electrophysiol..

[24] Koruth J.S., Kuroki K., Kawamura I., Brose R., Viswanathan R., Buck E.D., Donskoy E., Neuzil P., Dukkipati S.R., Reddy V.Y. (2020). Pulsed Field Ablation Versus Radiofrequency Ablation: Esophageal Injury in a Novel Porcine Model. Circ. Arrhythm. Electrophysiol..

[25] Reddy V.Y., Neuzil P., Koruth J.S., Petru J., Funosako M., Cochet H., Sediva L., Chovanec M., Dukkipati S.R., Jais P. (2019). Pulsed Field Ablation for Pulmonary Vein Isolation in Atrial Fibrillation. J. Am. Coll. Cardiol..

[26] Verma A., Boersma L., Haines D.E., Natale A., Marchlinski F.E., Sanders P., Calkins H., Packer D.L., Hummel J., Onal B. (2020). First-in-Human Experience and Acute Procedural Outcomes Using a Novel Pulsed Field Ablation System: The PULSED AF Pilot Trial. Circ. Arrhythm. Electrophysiol..

[27] Reddy V.Y., Petru J., Funasako M., Kopriva K., Hala P., Chovanec M., Janotka M., Kralovec S., Neuzil P. (2022). Coronary Arterial Spasm during Pulsed Field Ablation to Treat Atrial Fibrillation. Circulation.

[28] Zhang C., Neuzil P., Petru J., Funasako M., Hala P., Kopriva K., Koruth J.S., Dukkipati S.R., Reddy V.Y. (2024). Coronary Artery Spasm during Pulsed Field vs. Radiofrequency Catheter Ablation of the Mitral Isthmus. JAMA Cardiol..

[29] Reinsch N., Füting A., Höwel D., Bell J., Lin Y., Neven K. (2022). Cerebral safety after pulsed field ablation for paroxysmal atrial fibrillation. Heart Rhythm..

[30] Serban T., Mannhart D., Abid Q.U., Höchli A., Lazar S., Krisai P., Bettelini A.S., Knecht S., Kühne M., Sticherling C. (2023). Durability of pulmonary vein isolation for atrial fibrillation: A meta-Analysis and systematic review. Europace.

[31] Castrejón-Castrejón S., Martínez Cossiani M., Jáuregui-Abularach M., Basterra Sola N., Ibáñez Criado J.L., Osca Asensi J., Roca Luque I., Moya Mitjans A., Quesada Dorador A., Hidalgo Olivares V.M. (2023). Multicenter prospective comparison of conventional and high-power short duration radiofrequency application for pulmonary vein isolation: The high-power short-duration radiofrequency application for faster and safer pulmonary vein ablation (POWER FAST III) trial. J. Interv. Card. Electrophysiol..

[32] Kottmaier M., Popa M., Bourier F., Reents T., Cifuentes J., Semmler V., Telishevska M., Otgonbayar U., Koch-Büttner K., Lennerz C. (2020). Safety and outcome of very high-power short-duration ablation using 70 W for pulmonary vein isolation in patients with paroxysmal atrial fibrillation. Europace.

[33] Kuck K.H., Brugada J., Fürnkranz A., Metzner A., Ouyang F., Chun K.R., Elvan A., Arentz T., Bestehorn K., Pocock S.J. (2016). Cryoballoon or radiofrequency ablation for paroxysmal atrial fibrillation. J. Cardiopulm. Rehabil. Prev..

[34] Andrade J.G., Champagne J., Dubuc M., Deyell M.W., Verma A., Macle L., Leong-Sit P., Novak P., Badra-Verdu M., Sapp J. (2019). Cryoballoon or Radiofrequency Ablation for Atrial Fibrillation Assessed by Continuous Monitoring: A Randomized Clinical Trial. Circulation.

[35] Turagam M.K., Neuzil P., Schmidt B., Reichlin T., Neven K., Metzner A., Hansen J., Blaauw Y., Maury P., Arentz T. (2023). Safety and Effectiveness of Pulsed Field Ablation to Treat Atrial Fibrillation: One-Year Outcomes from the MANIFEST-PF Registry. Circulation.

[36] Reddy V.Y., Gerstenfeld E.P., Natale A., Whang W., Cuoco F.A., Patel C., Mountantonakis S.E., Gibson D.N., Harding J.D., Ellis C.R. (2023). Pulsed Field or Conventional Thermal Ablation for Paroxysmal Atrial Fibrillation. N. Engl. J. Med..

[37] Verma A., Haines D.E., Boersma L.V., Sood N., Natale A., Marchlinski F.E., Calkins H., Sanders P., Packer D.L., Kuck K.H. (2023). Pulsed Field Ablation for the Treatment of Atrial Fibrillation: PULSED AF Pivotal Trial. Circulation.

[38] Duytschaever M., De Potter T., Grimaldi M., Anic A., Vijgen J., Neuzil P., Van Herendael H., Verma A., Skanes A., Scherr D. (2023). Paroxysmal Atrial Fibrillation Ablation Using a Novel Variable-Loop Biphasic Pulsed Field Ablation Catheter Integrated with a 3-Dimensional Mapping System: 1-Year Outcomes of the Multicenter inspIRE Study. Circ. Arrhythm. Electrophysiol..

[39] Reddy V.Y., Anter E., Peichl P., Rackauskas G., Petru J., Funasako M., Koruth J.S., Marinskis G., Turagam M., Aidietis A. (2024). First-in-human clinical series of a novel conformable large-lattice pulsed field ablation catheter for pulmonary vein isolation. Europace.

[40] Della Rocca D.G., Marcon L., Magnocavallo M., Menè R., Pannone L., Mohanty S., Sousonis V., Sorgente A., Almorad A., Bisignani A. (2024). Pulsed electric field, cryoballoon, and radiofrequency for paroxysmal atrial fibrillation ablation: A propensity score-matched comparison. Europace.

[41] Maurhofer J., Kueffer T., Madaffari A., Stettler R., Stefanova A., Seiler J., Thalmann G., Kozhuharov N., Galuszka O., Servatius H. (2024). Pulsed-field vs. cryoballoon vs. radiofrequency ablation: A propensity score matched comparison of one-year outcomes after pulmonary vein isolation in patients with paroxysmal atrial fibrillation. J. Interv. Card. Electrophysiol..

[42] Badertscher P., Weidlich S., Knecht S., Stauffer N., Krisai P., Voellmin G., Osswald S., Sticherling C., Kühne M. (2023). Efficacy and safety of pulmonary vein isolation with pulsed field ablation vs. novel cryoballoon ablation system for atrial fibrillation. Europace.

[43] Rattka M., Mavrakis E., Vlachopoulou D., Rudolph I., Kohn C., Bohnen J., Yahsaly L., Siebermair J., Wakili R., Jungen C. (2024). Pulsed field ablation and cryoballoon ablation for pulmonary vein isolation: Insights on efficacy, safety and cardiac function. J. Interv. Card. Electrophysiol..

[44] Schipper J.H., Steven D., Lüker J., Wörmann J., van den Bruck J.H., Filipovic K., Dittrich S., Scheurlen C., Erlhöfer S., Pavel F. (2023). Comparison of pulsed field ablation and cryoballoon ablation for pulmonary vein isolation. J. Cardiovasc. Electrophysiol..

[45] Urbanek L., Bordignon S., Schaack D., Chen S., Tohoku S., Efe T.H., Ebrahimi R., Pansera F., Hirokami J., Plank K. (2023). Pulsed Field Versus Cryoballoon Pulmonary Vein Isolation for Atrial Fibrillation: Efficacy, Safety, and Long-Term Follow-up in a 400-Patient Cohort. Circ. Arrhythm. Electrophysiol..

[46] Reinsch N., Füting A., Hartl S., Höwel D., Rausch E., Lin Y., Kasparian K., Neven K. (2024). Pulmonary Vein Isolation Using Pulsed Field Ablation versus High-Power-Short-Duration Radiofrequency Ablation for Paroxysmal Atrial Fibrillation: Efficacy, Safety, and Long-Term Follow-up. Europace.

[47] Takagi T., Derval N., Duchateau J., Chauvel R., Tixier R., Marchand H., Bouyer B., André C., Kamakura T., Krisai P. (2023). Gaps after linear ablation of persistent atrial fibrillation (Marshall-PLAN): Clinical implication. Heart Rhythm..

[48] Chae S., Oral H., Good E., Dey S., Wimmer A., Crawford T., Wells D., Sarrazin J.F., Chalfoun N., Kuhne M. (2007). Atrial Tachycardia After Circumferential Pulmonary Vein Ablation of Atrial Fibrillation. Mechanistic Insights, Results of Catheter Ablation, and Risk Factors for Recurrence. J. Am. Coll. Cardiol..

[49] Ouyang F., Ernst S., Vogtmann T., Goya M., Volkmer M., Schaumann A., Bänsch D., Antz M., Kuck K.H. (2002). Characterization of reentrant circuits in left atrial macroreentrant tachycardia: Critical isthmus block can prevent atrial tachycardia recurrence. Circulation.

[50] Sawhney N., Anousheh R., Chen W., Feld G.K. (2010). Circumferential pulmonary vein ablation with additional linear ablation results in an increased incidence of left atrial flutter compared with segmental pulmonary vein isolation as an initial approach to ablation of paroxysmal atrial fibrillation. Circ. Arrhythm. Electrophysiol..

[51] Reddy V.Y., Anter E., Rackauskas G., Peichl P., Koruth J.S., Petru J., Funasako M., Minami K., Natale A., Jais P. (2020). Lattice-Tip Focal Ablation Catheter That Toggles Between Radiofrequency and Pulsed Field Energy to Treat Atrial Fibrillation: A First-in-Human Trial. Circ. Arrhythm. Electrophysiol..

[52] Anter E., Mansour M., Nair D.G., Sharma D., Taigen T.L., Neuzil P., Kiehl E.L., Kautzner J., Osorio J., Mountantonakis S. (2024). Dual-energy lattice-tip ablation system for persistent atrial fibrillation: A randomized trial. Nat. Med..

[53] Kim M.Y., Sandler B.C., Sikkel M.B., Cantwell C.D., Leong K.M., Luther V., Malcolme-Lawes L., Koa-Wing M., Ng F.S., Qureshi N. (2020). Anatomical Distribution of Ectopy-Triggering Plexuses in Patients with Atrial Fibrillation. Circ. Arrhythmia Electrophysiol..

[54] Markides V., Schilling R.J., Ho S.Y., Chow A.W., Davies D.W., Peters N.S. (2003). Characterization of left atrial activation in the intact human heart. Circulation.

[55] Bisignani A., Pannone L., Miraglia V., Sieira J., Iacopino S., Bala G., Ströker E., Overeinder I., Almorad A., Gauthey A. (2022). Feasibility and safety of left atrial posterior wall isolation with a new Cryoballoon technology in patients with persistent atrial fibrillation. PACE—Pacing Clin. Electrophysiol..

[56] Reddy V.Y., Anic A., Koruth J., Petru J., Funasako M., Minami K., Breskovic T., Sikiric I., Dukkipati S.R., Kawamura I. (2020). Pulsed Field Ablation in Patients with Persistent Atrial Fibrillation. J. Am. Coll. Cardiol..

[57] Karakasis P., Theofilis P., Vlachakis P.K., Korantzopoulos P., Patoulias D., Antoniadis A.P., Fragakis N. (2025). Atrial Fibrosis in Atrial Fibrillation: Mechanistic Insights, Diagnostic Challenges, and Emerging Therapeutic Targets. Int. J. Mol. Sci..

[58] Chelu M.G., King J.B., Kholmovski E.G., Ma J., Gal P., Marashly Q., AlJuaid M.A., Kaur G., Silver M.A., Johnson K.A. (2018). Atrial fibrosis by late gadolinium enhancement magnetic resonance imaging and catheter ablation of atrial fibrillation: 5-year follow-up data. J. Am. Heart Assoc..

[59] Marrouche N.F., Wazni O., McGann C., Greene T., Dean J.M., Dagher L., Kholmovski E., Mansour M., Marchlinski F., Wilber D. (2022). Effect of MRI-Guided Fibrosis Ablation vs. Conventional Catheter Ablation on Atrial Arrhythmia Recurrence in Patients with Persistent Atrial Fibrillation: The DECAAF II Randomized Clinical Trial. JAMA.

[60] Chen H., Li C., Han B., Xiao F., Yi F., Wei Y., Jiang C., Zou C., Shi L., Ma W. (2023). Circumferential Pulmonary Vein Isolation with vs. Without Additional Low-Voltage-Area Ablation in Older Patients with Paroxysmal Atrial Fibrillation: A Randomized Clinical Trial. JAMA Cardiol..

[61] Huo Y., Gaspar T., Schönbauer R., Wójcik M., Fiedler L., Roithinger F.X., Martinek M., Pürerfellner H., Kirstein B., Richter U. (2022). Low-Voltage Myocardium-Guided Ablation Trial of Persistent Atrial Fibrillation. NEJM Evidence.

[62] Ahn H.J., Lee S.R., Lee K.Y., Choi J.M., Kwon S., Choi E.K., Oh S. (2023). Left atrial fibrosis-guided ablation in patients with atrial fibrillation: A systematic review and meta-analysis of randomized trials. Eur. Heart J..

[63] Rivera A., Gewehr D.M., Braga M.A.P., Carvalho P.E.P., Ternes C.M.P., Pantaleao A.N., Hincapie D., Serpa F., Romero J.E., d’Avila A. (2024). Adjunctive low-voltage area ablation for patients with atrial fibrillation: An updated meta-analysis of randomized controlled trials. J. Cardiovasc. Electrophysiol..

[64] Verma A., Wazni O.M., Marrouche N.F., Martin D.O., Kilicaslan F., Minor S., Schweikert R.A., Saliba W., Cummings J., Burkhardt J.D. (2005). Pre-existent left atrial scarring in patients undergoing pulmonary vein antrum isolation: An independent predictor of procedural failure. J. Am. Coll. Cardiol..

[65] Younis A., Zilberman I., Krywanczyk A., Higuchi K., Yavin H.D., Sroubek J., Anter E. (2022). Effect of Pulsed-Field and Radiofrequency Ablation on Heterogeneous Ventricular Scar in a Swine Model of Healed Myocardial Infarction. Circ. Arrhythm. Electrophysiol..

[66] Kawamura I., Reddy V.Y., Santos-Gallego C.G., Wang B.J., Chaudhry H.W., Buck E.D., Mavroudis G., Jerrell S., Schneider C.W., Speltz M. (2023). Electrophysiology, Pathology, and Imaging of Pulsed Field Ablation of Scarred and Healthy Ventricles in Swine. Circ. Arrhythm. Electrophysiol..

[67] Gardziejczyk P., Wlazłowska-Struzik E., Skowrońska M., Baran J. (2023). Pulsed-field ablation using pentaspline catheter as a bail-out strategy for perimitral flutter related to the left atrium anterior wall scar. Hear. Case Rep..

[68] Mohanty S., Torlapati P.G., Casella M., Della Rocca D.G., Schiavone M., Doty B., La Fazia V.M., Pahi S., Pierucci N., Valeri Y. (2024). Redefining the blanking period after pulsed-field ablation in patients with atrial fibrillation. Heart Rhythm.

